# Variability of Jump Kinetics Related to Training Load in Elite Female Basketball

**DOI:** 10.3390/sports5040085

**Published:** 2017-11-04

**Authors:** Jan Legg, David B. Pyne, Stuart Semple, Nick Ball

**Affiliations:** 1 Strength and Conditioning, Australian Institute of Sport, Bruce 2617, Australia; david.pyne@ausport.gov.au; 2 Research Institute for Sport and Exercise, University of Canberra, Bruce 2617, Australia; Stuart.Semple@canberra.edu.au (S.S.); Nick.Ball@canberra.edu.au (N.B.)

**Keywords:** countermovement jump, variability, basketball

## Abstract

The purpose of this study was to quantify changes in jump performance and variability in elite female basketballers. Junior and senior female representative basketball players (*n* = 10) aged 18 ± 2 years participated in this study. Countermovement jump (CMJ) data was collected with a Gymaware™ optical encoder at pre-, mid-, and post-season time points across 10 weeks. Jump performance was maintained across the course of the full season (from pre to post). Concentric peak velocity, jump height, and dip showed the most stability from pre- to post-season, with the %CV ranging from 5.6–8.9%. In the period of the highest training load (mid-season), the variability of within-subject performance was reduced by approximately 2–4% in all measures except for jump height. Altered jump mechanics through a small (0.26 effect size) increase in dip were evident at mid-season, suggesting that CMJ analysis is useful for coaches to use as an in-season monitoring tool. The highest coefficient of variation (8–22%CV) in inter-set scores in all measures except eccentric peak velocity also occurred mid-season. It appears that in-season load not only impairs jump performance, but also movement variability in basketball players.

## 1. Introduction

Vertical jump performance has been studied extensively in male basketball players as an indicator of lower limb power, with more elite players recording greater jump heights [1]. Time on court correlates highly with the anaerobic performance of vertical jump height, speed, and agility, indicating that physical capabilities play a strong role in team selection [2]. The countermovement jump (CMJ) is considered a practical assessment tool in elite sports to examine kinetic and or kinematic performance variables [3,4].

The ability to produce force is essential for jumping ability in basketball players, with jumping considered an acceptable measure for evaluating the stretch shortening cycle (SSC) [5,6]. Muscle function and the ability to quickly transition from eccentric to concentric contractions via the SSC is critical to many offensive and defensive manoeuvres performed in basketball, including rebounding, shooting, and sprinting. Analysis of an athlete’s SSC can also be useful in monitoring the effects of fatigue on performance [7]. This type of analysis has not been conducted previously on elite female basketball players across a competition season.

The use of a linear position transducer (LPT) by high-performance coaches and their support staff to measure CMJ performance is increasingly common. An LPT provides a portable and effective method of analysing the displacement of the bar or body [8]. Kinematic data from LPTs is differentiated to estimate force and power when subject mass is factored in, with strong relative validity compared with a force plate [8,9]. Until recently, analysis of CMJ data has been limited to values relating to the concentric phase of the jump, such as jump height and peak power. However, the importance of monitoring eccentric jump variables as a result of altered jumping mechanics after training adaptations or fatigue is now recognized [10,11]. CMJ testing can also provide valuable insights into neuromuscular fatigue, response to training loads, and subsequent recovery in high-performance sport environments [3]. This information can be collated with subjective internal training load data to give a comprehensive picture of the athlete’s preparation and response to training [12].

Countermovement jump analysis can provide worthwhile information, but it is imperative that coaches understand the typical variation or repeatability of the testing. It is critical for coaches to understand where a meaningful change in performance has occurred or whether the magnitude of changes lie within the normal reproducibility of the outcome variable (jump performance), known as the typical error [13]. The level of expertise an athlete has acquired in the performance skill will affect reliability, with elite athletes having a demonstrated ability to achieve outcome performances more consistently [14]. This is despite dynamical systems theory and coordination profiling that proposes that as an athlete’s skill level and expertise improves the motor system variability will increase in order to achieve these consistent performance outcomes [15]. These theories propose that skilled performers adapt to their surroundings and any unique constraints (environmental, task, and organismic) to achieve stable task execution through movement variability. This adaptation mechanism may only be applicable to open sport skills such as shooting in basketball when under pressure from an opposing player rather than the closed skill of a CMJ, particularly in developing athletes [16,17]. Consequently, whilst kinetic variability is likely to be relatively stable in a CMJ, this has yet to be demonstrated.

Acceptable within-subject reliability for force-related measures of the CMJ has been defined as a coefficient of variation (%CV) between 2.8 and 9.5% for single trials, and between 0.8 and 6.2% when six trials are used [3,18]. The error rate for a six-jump protocol is estimated between 1.1–3.2% for most kinetic and kinematic variables, except for rate of force development, which may be as high as 13–16% [3]. While studies have focused on the magnitude of training interventions [10,19], the changes in within-subject reliability of the kinetic profile from the CMJ in elite female basketball players across the course of a training period is unclear. Research is required to determine if kinetic variability can be decreased as a function of improved performance in basketball players.

The purpose of this study was to quantify the pattern of within-subject variability in jump height and kinetic variables in elite female basketball players over the course of a competitive season. This investigation also sought to determine if performance increases in jump height and kinetic variables were evident within the 10-week in-season training period. This information should provide valuable insight to coaches by highlighting the specific areas athletes can target in training to improve power production and jump performance. We hypothesized that while jump performance may not improve substantially in-season, the within-subject variability of SSC parameters would likely decrease as a result of structured training throughout the competition phase.

## 2. Materials and Methods

### 2.1. Experimental Approach to the Problem

Ten female Basketball Australia Centre of Excellence scholarship holders completed a ten-week in-season strength and conditioning program to improve jump technique and performance. A single-group longitudinal assessment was employed over the competition season. Kinetic variables were recorded from the CMJ at three different time points across the season (pre-, mid-, and post-season) with within-subject and between-subject reliability analysis undertaken to assess kinetic variability.

### 2.2. Participants

Australian female junior and senior representative basketball players (*n* = 10) aged 18 ± 2 years, height 1.85 ± 0.09 m, mass 75.2 ± 7.2 kg, sum of seven skinfolds 82.6 ± 13.9 mm participated in this study. All players performed jump testing as part of their usual training program during a designated afternoon training session. All participants trained together and represented the same team throughout the competition. Informed consent was given by all participants, with ethics approval granted through the Australian Institute of Sport Ethics Committee. Players were excluded from individual testing sessions if they were not currently completing a full training load for basketball due to injury, fatigue, or illness. The participants had a range of resistance training experience with 12 months (*n* = 6), 2 years (*n* = 2), and 6 years (*n* = 2).

### 2.3. Procedures

Resistance Training and Training Load. Athletes were required to complete their usual periodized resistance training sessions consisting of 2–3 full body sessions per week (depending on scheduling of games). The distribution of exercise selection and consequent loading across each training week was stability and control 20%, power 30–40%, and strength 40–50%. Mean weekly training load consisted of three full-team on-court training sessions of 2–2.5 h duration, two individual high-intensity on-court training sessions of 30 min duration, and two low-intensity 60 min shooting sessions. This load was elevated at the mid-season point during an intensive training camp of 5 days duration to a mean of 4 h of court-based team training daily—approximately a two-fold increase (doubling) in duration. Rating of perceived exertion (RPE) was collected following each training session as well as games on the Borg 10 point scale [20]. Athletes were required to identify the RPE for each session, and this was multiplied by the session duration. The total training load using the session-RPE method for each week at the pre-, mid-, and post-season was then calculated as the total load sum of sessional data for each athlete aggregated as a group mean. Match loads were included in the total training load as minutes played multiplied by the RPE score. All participants were familiar with using RPE as part of their regular load monitoring of training and games.

Jump Tests. Countermovement jump data was collected with a Gymaware™ optical encoder (50 Hz sample period with variable rate sampling with level crossing detection to capture data points; Kinetic Performance Technology, Canberra, Australia) [21]. The Gymaware™ optical encoder was attached via a tether to the right side of a 0.3 kg wooden bar. Bar placement on the back for each subject was between the superior portion of the scapula and vertebra C7. The stance for each subject was constrained to within 15 cm of the lateral portion of the individual’s deltoid, as specified by McBride et al. [22]. Participants initiated the CMJ via a downward countermovement to a self-selected depth, followed immediately by a maximal effort vertical jump. Participants were instructed to keep constant downward pressure on the bar throughout the jump and encouraged to reach a maximum jump height with every trial in an attempt to maximize power output. Participants were encouraged and reminded between trials to “jump high” and “jump fast”. Each subject performed five trials, with a small pause between each trial to steady themselves and stand tall. The following variables were collected for normalised power: peak power (W) and concentric (Conc) mean power (W). Mean watts (W/kg) and peak watts (W/kg) were used to determine absolute power. Concentric peak velocity (m/s), eccentric peak velocity (m/s), as well as jump height (cm) and dip (cm) were also recorded. 

Prior to jump testing, all athletes completed the same 10-min warm up consisting of dynamic flexibility work followed by 10 bodyweight squats, three bodyweight CMJs at subjective intensity of 60% maximal effort, and three bodyweight CMJs at 90% maximal effort. All athletes had previously completed this type of jump testing as part of their regular training for at least 4 weeks prior to the investigation.

### 2.4. Statistical Analyses

Means and standard deviations (SD) were calculated for the kinetic and kinematic variables at pre-season, mid-season, and post season. Independent variables were the time point of the testing (pre/mid/post), while the dependent variables were power, velocity, jump height, and dip. The effect of training on jump performance was quantified by determining the mean change in test scores over the 10-week training period using an independent student’s *t*-test. Precision of estimation was expressed as the 90% confidence limits. A standardized mean effect was used to characterize the magnitude of change. Magnitudes of standardized effects were interpreted against the following criteria: trivial 0.0, small 0.2, moderate 0.6, and large 1.0 [13]. An effect was deemed unclear if its confidence limits simultaneously overlapped the thresholds for a substantially positive and negative change.

To examine the mean within-subject variability in jump performance and kinetics at baseline and changes in variability across the season, we computed the % coefficient of variation (%CV). To compare the magnitude of change in variation for phase of the season we divided the %CV for consecutive pairs of testing sessions. Ratios within a range of 0.87 to 1.15 were considered trivial; a ratio >1.15 indicated that CMJ performance was substantially more variable, whereas a ratio <0.87 indicated that test results were substantially less variable [23].

To examine the mean between-subject variability in jump performance and kinetics at baseline and changes in variability across the season, we calculated an intraclass correlation coefficient (ICC). We interpreted the magnitude of the correlation using the thresholds of 0.20 (low), 0.50 (moderate), 0.75 (high), 0.90 (very high), and 0.99 (extremely high) consistency [13]. A difference >0.10 in the correlation coefficient between time points across the season was deemed substantial.

## 3. Results

No substantial changes in jump performance occurred from pre- to post-season (Table 1), with jump height performance measures stable at all measurement points across the season. A moderate reduction in watts/kg and a small reduction in concentric mean power was evident from the pre-season to mid-season. However, these effects were reversed by the end of the season, with a moderate and small increase in these measures from the mid-season to post-season. There were decreased values for %CV, suggesting more consistent jumping, from the beginning of the season to mid-season (which coincides with the period of the highest training loads) in all variables except jump height.

Table 2 details the inter-set reliability across the season, with mid-season showing the highest variation in executing five CMJ in all variables except eccentric peak velocity, dip, and height, which remained stable. Eccentric peak velocity also had a large increase in variability in the post-season testing, exhibiting a within-set %CV of 46%; however, this change was deemed unclear. There were substantial improvements in within-set reliability for mean watts (W/kg) and mean power from pre-season to post-season testing. Concentric peak velocity, jump height, and dip showed the greatest consistency from pre- to post-season, with the CV ranging from 6–10%. However concentric peak velocity was less reliable mid-season, with an increase in %CV and a substantial reduction in reliability from moderate to low.

Group training loads were recorded to be the following from RPE scores: pre-season 3195 ± 1083 (arbitrary units; mean ± SD), mid-season 4344 ± 1376, and post-season 2161 ± 1043. The groups’ training loads were markedly higher during mid-season, corresponding with high reported RPE values.

## 4. Discussion

Training during a ten-week in-season competition phase did not elicit substantial changes in jump performance in a cohort of elite female basketball players. While kinetic performance of relative (moderate) and mean (small) concentric power declined from pre- to mid-season, these measures were restored by the post-season. Consistency of jumping (reliability) in the context of performing a standard five jump CMJ protocol was improved across the course of the season for mean watts/kg, mean power, concentric peak velocity, jump height, and dip. These outcomes indicate that while performance markers such as height and power did not improve, consistency of jump performance improved as a result of a structured training program throughout the competition phase. Within-subject variation decreased across the season in mean power and dip, whereas height and concentric peak velocity remained steady, demonstrating increased reliability as well as consistency in the primary performance outcomes.

The ability to efficiently produce force is essential for jumping ability for basketball players, with the propulsive action considered to be an acceptable measure for evaluating explosive characteristics [5,6]. In the protocols used in this investigation (i.e., restricting arm swing), jump height values are lower than those reported in other investigations that permitted a more natural jumping action [1]. Concentric peak velocity, jump height, and dip showed the greatest consistency from pre- to post-season, with the CV ranging from 6–10%, which may be attributed to the established relationship between jump height and velocity [24]. The concentric peak velocity of the players in the pre-season of this investigation (3.22 ± 0.25 m/s) was comparable to other sports of similar age and performance (women’s football 3.00 ± 0.20m/s and netball 2.80 ± 0.20 m/s) [25]. Concentric peak velocity was less reliable mid-season, with an increase in the CV% and a marked reduction in the intra-set consistency from moderate to low reliability. This reduction may relate to altered jumping mechanics of the athletes during this fatigued training period, as dip was increased to maintain jump height despite more variable velocity measures. The altered jumping mechanics may also explain the large changes in the power characteristics observed in the peak power, peak watts, and concentric mean power as the athletes modified their jump in order to achieve the performance outcome of jump height.

The improved consistency of jumping in this squad may be attributed to improvements in coordination, control and skill as the season progressed. The likely explanation is improvement in jumping motor patterns. Given the limited experience of strength training in the majority of the squad, motor learning as described by Newell would most likely have occurred throughout the competition phase [26]. Strength and power levels can increase significantly for athletes trained in a supervised environment, when compared to those training in an unsupervised environment [27]. An increase in motor control and coordination of jumping skills would be anticipated in athletes training for the first time within a centralized training environment under direct coaching and supervision [28]. It is likely that the athletes in this investigation were beginning to master the skill of jumping, as demonstrated by the ability to perform more consistently [14]. This outcome follows traditional motor learning principles that once skilled performance has been acquired there is likely a concomitant reduction in coordination variability. However, the changed dip patterns seen in this squad following fatigue raises the question of whether coordination variability in athletes is influenced by both adaptation and fatigue.

With increased training loads during mid-season (4344 ± 1376) compared to pre-season (3195 ± 1083), the magnitude of the dip was increased across the squad, suggesting that the athletes were completing a deeper squat prior to commencing the jump in order to generate the necessary power to achieve a similar jump height. This outcome may be explained by the concept that skilled performers are able to demonstrate increased movement variability and altered movement strategies to achieve consistent performance outcomes [5,11]. Impulse was likely to have been altered in this fatigued state, with time under the force-time curve increased [29]. This effect is likely related to athletes inherently trying to generate more force through increased eccentric loading when in a fatigued state, although only a trivial positive increase in peak power and peak watts was observed from pre- to mid-season. Elite snowboard cross-athletes exhibited a similar change in jump mechanics when athletes were fatigued with increased dip in an effort to maintain jump height [11]. While neuromuscular fatigue appears to have altered the biomechanics of the CMJ through increased dip measures in both Gathercoleet al.’s research and this investigation, no significant decreases in capacity as measured by jump height were evident [11]. This outcome may be explained by the motor learning concept that in well-learned movements, consistent performance outcome is often associated with high intra-limb joint coordination variability whereby the movement pattern an athlete uses may appear different but the end result is the same [30].

When training loads based on RPE were at their highest point (mid-season), the within-group variation in selected kinetic variables was reduced. It might be the case that neuromuscular fatigue limits the jumping capacity of the best overall jumpers and brings them to similar levels of lesser-skilled jumpers within the group. Neural control as demonstrated through coordination could be compromised following fatigue and affect the strength available for optimal jump performance [31]. The change in mean scores of peak power was increased mid-season in this squad, along with small (−0.36 and −0.26) decrements of the within-subject strength-based variables of relative peak watts (W/kg) and concentric mean power. Over the course of this investigation, strength markers were not able to be collected, but these results are likely related to a reduction in maximum strength driven by neuromuscular fatigue at the mid-point of the season. A reduction in strength has been linked to decreased jumping ability in numerous sports [22,32].

This investigation did not show any significant changes in jump performance within the 10-week in-season training block. The stability of jump height in this in this study is in contrast to a 7-week in-season competition phase for junior male rugby league players that detailed accumulating fatigue and reduced CMJ performance [7]. Reductions in CMJ performance was also reported in division 1 collegiate male soccer players who exhibited a 13.8% reduction in vertical jump across an 11-week season [33]. While fatigue was evident in this investigation at the mid-season point as demonstrated by impaired jump mechanics, recovery was sufficient throughout the season to avoid any significant decreases in jump performance.

## 5. Conclusions

During the course of a competitive season, basketball players are exposed to a combination of the rigors of games and training demands both on the court and in the weight room. Strength and conditioning coaches should consider monitoring movement variability of the CMJ to assess training effects as well as the degree of neuromuscular fatigue. This investigation showed increased movement variability in the CMJ when training loads were at their peak along with increased fatigue. While the commonly investigated performance marker of jump height appears stable even when players are in a fatigued state, strength and conditioning coaches can monitor eccentric jump variables for signs of overtraining or overreaching. An increase in eccentric duration has the potential to negatively affect sport outcomes. For example, a longer duration spent achieving maximal push off on the basketball court could lead to a missed pass or rebound. Given the importance of movement speed and mechanical efficiency, these results could affect the training load prescribed to athletes during a competition season.

## Figures and Tables

**Table 1:** Changes in within-subject lower body power characteristics in women’s basketball players across a season using a countermovement jump. %CV coefficient of variation; pre-season; mid-season, post-season. ∆X: change in the mean; mod: moderate; Ecc: eccentric; Conc: concentric.

	Pre-Season		Mid-Season		Change (Pre vs. Mid)		Post-Season		Change (Pre vs. Post)		Change (Mid vs. Post)	
	Mean	%CV	Mean	%CV	∆X	Effect Size	Mean	%CV	∆X	Effect Size	∆X	Effect Size
Mass (kg)	76.2 ± 7.6		76.3 ± 8.3				76.4 ± 7.8					
Mean Watts (W/kg)	43.0 ± 5.1	12	41.1 ± 4.4	11	−2.0	−0.36	43.2 ± 7.2	17	0.17	0.00	2.13	0.35
						mod				trivial		mod
Peak Power (W)	5130 ± 862	17	5160 ± 651	13	30	0.06	5039 ± 889	18	−91	−0.10	−121	−0.16
						trivial				trivial		trivial
Peak Watts (W/kg)	67.2 ± 9.6	14	67.8 ± 6.8	10	0.18	0.05	66.3 ± 12.2	18	−2.0	−0.17	−1.5	−0.22
						trivial				trivial		small
Conc Mean Power (W)	3275 ± 592	18	3129 ± 422	14	−171	−0.26	3269 ± 499	15	−30	−0.03	140	0.23
						small				trivial		small
Conc Peak Velocity (m/s)	3.22 ± 0.25	8	3.23 ± 0.19	6	−0.02	−0.05	3.23 ± 0.24	7	−0.02	−0.08	0.01	−0.03
						trivial				trivial		trivial
Height (m)	0.39 ± 0.05	13	0.40 ± 0.07	17	0.004	0.00	0.39 ± 0.06	15	−0.01	−0.18	−0.01	−0.18
						trivial				trivial		trivial
Dip (m)	0.43 ± 0.11	27	0.46 ± 0.11	25	0.013	0.26	0.44 ± 0.12	29	−0.004	−0.02	−0.02	−0.21
						small				trivial		small
Ecc Peak Velocity (m/s)	1.0 ± 0.2	23	0.9 ± 0.2	19	−0.07	−0.09	1.0 ± 0.2	22	−0.05	−0.04	0.02	0.05
						trivial				trivial		trivial

**Table 2:** Inter-set reliability of performing five countermovement jumps (CMJs) at each time point presented as %CV, 90% confidence interval, and the intraclass correlation coefficient (ICC).

	Pre-Season	ICC	Mid-Season	ICC	Post-Season	ICC
Mean Watts (w/kg)	12, 9–22	0.46	14, 11–27	0.29	9, 7–17	0.69
Peak Power (W)	15, 11–27	0.62	18, 14–34	0.22	15, 12–28	0.50
Peak Watts (w/kg)	15, 11–27	0.45	18, 14–34	0.05	15, 12–28	0.54
Mean Power (W)	12, 9–22	0.64	14, 11–27	0.46	9, 7–17	0.69
Conc Peak Velocity (m/s)	6, 5–11	0.62	8, 6–15	0.17	6, 4–10	0.60
Height (cm)	7, 5–11	0.79	8, 6–15	0.84	6, 5–11	0.87
Dip (cm)	9, 7–15	0.93	10, 7–17	0.89	7, 5–12	0.94
Eccentric Peak Velocity (m/s)	27, 24–67	0.58	22, 18–50	0.59	46, 43–138	0.43

Thresholds for ICC: 0.20 (low), 0.50 (moderate), 0.75 (high), 0.90 (very high), and 0.99 (extremely high) consistency.
